# A bidirectional association between smartphone addiction and depression among college students: A cross-lagged panel model

**DOI:** 10.3389/fpubh.2023.1083856

**Published:** 2023-01-24

**Authors:** Kexin Zhang, Haiyun Guo, Tianli Wang, Jianghui Zhang, Guojing Yuan, Juan Ren, Xueqing Zhang, Huayu Yang, Xiaoyan Lu, Zhihui Zhu, Jun Du, Haiyan Shi, Guifang Jin, Jiahu Hao, Ying Sun, Puyu Su, Zhihua Zhang

**Affiliations:** ^1^Department of Epidemiology and Biostatistics, School of Public Health, Anhui Medical University, Hefei, China; ^2^Hefei Center for Disease Control and Prevention, Hefei, China; ^3^The Second Hospital of Anhui Medical University, Hefei, China; ^4^Department of Maternal, Child, and Adolescent Health, School of Public Health, Anhui Medical University, Hefei, China

**Keywords:** smartphone addiction, depression, college students, longitudinal study, cross-lagged panel model (CLPM)

## Abstract

**Background:**

Smartphone addiction (SA) is associated with adverse consequences, especially for freshmen. Evidence indicates that SA is associated with depression, and it is necessary to conduct a longitudinal study to explore the association further.

**Methods:**

SA (measured by the Smartphone Addiction Scale-Short Version) and depression (measured by the Zung's Self-Rating Depression Scale) among 1,186 freshmen were surveyed at baseline and a respective 12-month follow-up for each participant. The application of a cross-lagged panel model approach (CLPM) revealed an association between SA and depression after adjusting for demographic variables.

**Results:**

The CLPM results showed a significant path from baseline SA to follow-up depression (β = 0.08, *P* < 0.001) and a significant path from baseline depression to follow-up SA (β = 0.08, *P* < 0.001). Compared with the overall cross-lagged model, the cross-lagged coefficient of the path from baseline SA to follow-up depression increased in the female group (β = 0.10, *P* = 0.015), and the cross-lagged coefficient of the path from baseline depression to follow-up SA also increased significantly (β = 0.15, *P* < 0.001). In contrast, the cross-lagged model in the male group showed no predictive effect between SA and depression (*P* > 0.05).

**Conclusions:**

The current study showed a significant bidirectional association between smartphone addiction and depression among freshmen, but only in the female population.

## 1. Introduction

Smartphone addiction (SA) is characterized by excessive attention and uncontrolled dedication to one's smartphone (1). It has been observed that SA has worryingly increased worldwide in recent years. Zhong et al. (2) conducted a systematic review and found that the prevalence of SA among Asian medical students was 41.93%. Meng et al. (3) found that the global pooled prevalence of SA was 34.5% among college and high school student populations over the last 2 years. In addition, numerous studies have shown that SA is associated with several psychiatric comorbidities, including depression, anxiety, autism spectrum disorder and suicide attempt (4–7). Depression is also common among adolescents. A recent systematic review reported a global prevalence of depression among adolescents of 34% (95% CI: 30–38%), with a higher prevalence among females than males (8). Depression can have serious adverse health consequences for adolescents and may increase the risk of suicide attempts, substance abuse, anxiety disorders, and comorbid somatic disorders (9). Freshmen are a particular population transitioning from high school to college, facing new physical and interpersonal environments, and prone to mental health problems (10). After leaving the high school environment with a heavy academic load and little free time and entering relatively free college life, freshmen also have more time to use smartphones. In addition, they may become heavily dependent on smartphones, either as a tool to stay in touch with family and friends or as a necessary tool for studying and living in a new environment (11).

Different studies have provided preliminary empirical support for the association between SA and depression. In cross-sectional studies, some suggest that SA may be a risk factor for depression (12, 13), while others suggest that depression may be a risk factor for SA (14, 15). Li et al. pooled 21 studies for meta-analysis and showed that SA was weakly to moderately positively associated with depression (*r*=0.36) (16). Several hypotheses or approaches have been proposed to explain the interrelationship between SA and depression. The first is the Compensatory Internet Use Theory (CIUT) (17). This theory states that individuals' use of smartphones is a means for them to cope with painful emotional states and to access social needs that may not be met in the real world (17). In this model, depressed individuals have difficulty feeling pleasure gained from social interactions but tend to use their phones more frequently as an adaptive coping mechanism in difficult situations and may be prone to SA (18, 19). The second is the upward social comparison hypothesis, which refers to people comparing themselves with those they perceive to be in a more favorable position (20). For this hypothesis, repeated exposure to idealized information during smartphone use may lower the user's self-esteem, trigger depression, and enhance depression over time (21). In addition, Samra et al. (22) noted that females used social media more problematically and compared themselves more negatively to others on social media than males. This suggests the need to pay attention to gender differences while exploring the relationship between SA and depression.

It has been reported that female students report a higher prevalence of depressive symptoms than male students (9). Crockett et al. (23) found that females have more depressive moods but fewer problems with concentration and psychomotor retardation/agitation than males. Albursan et al. (24) indicated that although female students were more likely to use smartphones, the effect of over-reliance on smartphones on academic achievement appears to be more pronounced among male students. Zhu et al. (25) found that depression predicted Internet addiction only among males but not among females, suggesting that the bidirectional predictive relationship between Internet addiction and depression may depend on gender. Considering that the above study found gender differences in addictive behaviors and depressive symptoms, it is necessary to further explore the role of gender in the longitudinal association of SA and depression.

Longitudinal survey analysis to test these hypotheses concerning the potential etiological association between SA and depression will undoubtedly be valuable. Zhou et al. (26) conducted a 6-month follow-up of 313 high school students and found that depression unidirectionally predicted SA. Chen et al. (27) conducted a 9-month follow-up of 308 Hong Kong university students and found that the growth of SA was positively associated with depression. Published longitudinal studies are limited in several ways, as follows, relatively small sample sizes, short follow-up intervals, no studies have focused on the stability of SA or depression, and no consideration of the role of gender as an essential factor in the relationship. In light of the limitations of published studies, a longitudinal study is warranted to explore the association between SA and depression further. The present study aims to test the hypothesized bidirectional association between smartphone addiction and depression among freshmen using the CLPM model and to explore the role of gender in this association.

## 2. Methods

### 2.1. Participants

This is a one-year prospective study. In September 2020, we recruited 1226 freshmen from a medical college in Hefei, Anhui Province, China. An electronic questionnaire was used, with the investigator providing a QR code between classes and students scanning the code with their smartphones to fill out the questionnaire. Among the included respondents, 1,186 (96.47%) also completely responded to the questions relevant to the present study in a respective follow-up survey after 12 months. The questionnaire completion was anonymous, and data from the two surveys were matched according to a unique code assigned to each student. Informed consent was obtained from every participant prior to two questionnaire surveys. The Research Ethics Committee has approved this study of the Anhui Medical University (No. 20190495).

### 2.2. Measures

The data collected in the present study included socio-demographics, living habits, and health conditions. The variables examined in the socio-demographic section included gender, age, and their parents' education level (bachelor degree or higher). The lifestyle factors included body-mass index (BMI), daily exercise time (hours), daily smartphone use time (hours), and sleep time per night (hours). The health conditions included smartphone addiction (SA) and depression severity, measured by the Smartphone Addiction Scale-Short Version (SAS-SV) (Supplementary Tables 1, 2) and the Zung's Self-Rating Depression Scale (SDS) (Supplementary Tables 3, 4), respectively.

The Smartphone Addiction Scale-Short Version (SAS-SV) consisted of 10 symptoms of excessive smartphone use (28). Six experts selected 10 items of this short version from the original 33-item SAS. Each item was scored on a six-point Likert scale, and a higher total score indicates a more severe level of smartphone addiction. The widely used SAS-SV cut-off scores of ≥31 for males and ≥33 for females were used as proposed by the scale developers (28). We used the version translated by Xiang et al., which has been shown in previous studies to have good reliability and validity of SAS-SV in Chinese populations (29). In this study, *Cronbach's* α for SAS-SV at baseline and follow-up surveys were 0.83 and 0.89, respectively. Confirmatory factor analysis showed an acceptable model fit of SAS-SV at the baseline survey (χ*2/df* = 12.83, CFI =0.87, GFI =0.92, and RMSEA = 0.10) and at the follow-up survey (χ*2/df* = 19.74, CFI =0.88, GFI =0.88, and RMSEA = 0.13).

Zung's Self-Rating Depression Scale (SDS) has been widely used to assess depression during the past week (30). It consists of 20 self-rated questions, each item rated on a 4-point scale ranging from 1 (a little of the time) to 4 (most of the time). The total score was acquired by multiplying the raw score by 1.25. A higher total score indicates a more severe level of depression. An SDS score of 50 (raw score = 40) suggests clinically significant symptoms (31). Many previous studies had used the Chinese versions of SDS (32) to evaluate the depression symptoms in Chinese population including adolescents (33). In this study, *Cronbach's* α for SDS at baseline and follow-up surveys were 0.80 and 0.84, respectively. Confirmatory factor analysis showed that the χ*2/df*, CFI, GFI, and RMSEA of SDS at baseline survey were 7.47, 0.75, 0.88, and 0.07, respectively; and the χ*2/df*, CFI, GFI, and RMSEA at follow-up survey were 19.08, 0.62, 0.68, and 0.12, respectively.

### 2.3. Statistical procedures

Descriptive characteristics were investigated at baseline between the responders and non-responders (Table 1). Categorical variables were reported as frequency (percentages), and continuous variables were reported as means (standard deviations).

**Table 1:** Descriptive characteristics comparing groups responding at baseline only, or both surveys.

	**Participants who responded to baseline survey only (*N* = 1226)**	**Participants who responded to both surveys (*N* = 1186)**	***P* value**
	**Mean (SD) or N (%)**	**Mean (SD) or N (%)**	
**Socio-demographics**			
Age, years^a^	18.08 (2.45)	18.08 (2.22)	0.979
Gender, male^b^	587 (47.9)	566 (47.7)	0.939
Parental education level (Bachelor's degree or above)^b^	273 (22.3)	262 (22.1)	0.917
Maternal education level (Bachelor's degree or above)^b^	176 (14.4)	170 (14.3)	0.988
**Living habits**			
Body-Mass Index^a^	21.63 (3.52)	21.64 (3.52)	0.948
Daily exercise time, hours^a^	1.17 (0.78)	1.17 (0.78)	0.931
Daily smartphone use time, hours^a^	3.77 (2.02)	3.76 (2.01)	0.885
Sleep time per night, hours^a^	7.18 (1.05)	7.18 (1.05)	0.970
**Health conditions**			
SAS-SV scores^a^	27.85 (7.63)	27.66 (7.31)	0.528
SDS scores^a^	41.50 (8.09)	41.62 (8.12)	0.732

Values are percentages for categorical variables, means for continuous variables and P value. SD, standard deviation. SAS-SV, Smartphone Addiction Scale-Short Version; SDS, Zung's Self-Rating Depression Scale.

a P-value calculated using the independent-samples t-test;

b P-value calculated using the Chi-square test.

Univariate differences in demographic variables frequencies and rates in individuals with SA and depression were tested with the Chi-square test. Adjusted odds ratios (ORs) and their 95% CIs for individuals with SA or depression and demographic groups were estimated using a binary logistic regression (Enter) model adjusted for confounding effects. For the binary logistic regression (Enter) model, the dependent variables were the status of SA or depression (Yes or No), and the independent variables were age, biological gender, parental education level, maternal education level, and BMI.

Furthermore, AMOS 26.0 was used to calculate structural equation models (SEM) in the cross-lagged panel model (CLPM) design, including the follow-up sample (n = 1,186) to examine possible bidirectional effects between SAS-SV scores and SDS scores. Model fit was evaluated using χ^2^ index (χ^2^*/df* ), comparative fit index (CFI), goodness-of-fit index (GFI), and root-mean-square error of approximation (RMSEA). Eventually, we performed a multi-group analysis by gender. Statistical significance was accepted at *P* < 0.05 in all analyses.

## 3. Results

### 3.1. Demographics

Characteristics between responders and non-responders of the study sample are presented in Table 1. There were 1,186 (96.74%) participants responding at follow-up. No significant differences were found between participants who responded to the baseline survey only and those who responded to both in terms of socio-demographics, living habits and health conditions. In view of this result, we focused mainly on reporting data from participants who responded to both surveys (*n* = 1,186) in the following sections.

### 3.2. The rates and associated factors of SA and depression at baseline and follow-up surveys

The rates of baseline SA and depression were 32.0% (95% CI: 29.4%, 34.7%) and 10.7% (95% CI: 8.9%, 12.5%), respectively; they increased to 52.5% (95% CI: 49.7%, 55.4%) and 28.2% (95% CI: 25.7%, 30.8%) at follow-up, respectively (Tables 2, 3).

**Table 2:** The detection rates and predictors for smartphone addiction and depression in the baseline among respondents who responded to both surveys.

	**Smartphone addiction**					**Depression**				
	***N***	**Prevalence rates** ** (95%CI^a^)**	***χ^2^* ^b^ (*P* value)**	**Adjusted OR^c^ (95%CI)**	***P* value**	***N***	**Prevalence** ** rates (95%CI^a^)**	***χ^2^* ^b^ (*P* value)**	**Adjusted OR^d^ (95%CI)**	***P* value**
Total (*n* = 1,186)	380	32.0 (29.4, 34.7)				127	10.7 (8.9, 12.5)			
**Gender**			22.00 (< 0.001)					4.76 (0.03)		
Male (*n* = 566)	219	38.7 (34.7, 42.7)		1.93 (1.49, 2.50)		49	8.7 (6.3, 11.0)		1	
Female (*n* = 620)	161	26.0 (22.5, 29.4)		1	< 0.001	78	12.6 (10.0, 15.2)		1.73 (1.16, 2.56)	0.01
**Parental education level**			1.80 (0.18)					1.88 (0.17)		
Below bachelor's degree (*n* = 924)	305	33.0 (30.0, 36.0)		1		105	11.4 (9.3, 13.4)		1	
Bachelor's degree or above (*n* = 262)	75	28.6 (23.1, 34.1)		0.90 (0.60, 1.35)	0.60	22	8.4 (5.0, 11.8)		1.10 (0.58, 2.09)	0.77
**Maternal education level**			2.26 (0.13)					2.76 (0.10)		
Below bachelor's degree (*n* = 1,016)	334	32.9 (30.0, 35.8)		1		115	11.3 (9.4, 13.3)		1	
Bachelor's degree or above (*n* = 170)	46	27.1 (20.3, 33.8)		0.94 (0.59, 1.50)	0.79	12	7.1 (3.2, 10.9)		0.63 (0.29, 1.37)	0.25
**Body-Mass Index**			0.86 (0.84)					4.46 (0.22)		
Wasting (*n* = 207)	62	30.0 (23.7, 36.2)		1		23	11.1 (6.8, 15.4)		1	
Normal (*n* = 720)	235	32.6 (29.2, 36.1)		1.09 (0.77, 1.54)	0.64	84	11.7 (9.3, 14.0)		1.07 (0.65, 1.76)	0.79
Overweight (*n* = 187)	58	31.0 (24.3, 37.7)		0.93 (0.60, 1.45)	0.75	17	9.1 (4.9, 13.2)		0.91 (0.46, 1.79)	0.78
Obese (*n* = 72)	25	34.7 (23.5, 46.0)		1.12 (0.62, 2.02)	0.70	3	4.2 (0.6, 8.9)		0.39 (0.11, 1.37)	0.14
**Smartphone addiction**								28.03 (< 0.001)		
No (*n* = 806)						60	7.4 (5.6, 9.3)		1	
Yes (*n* = 380)						67	17.6 (13.8, 21.5)		2.89 (1.97, 4.24)	< 0.001
**Depression**			28.03 (< 0.001)							
No (*n* = 1,059)	313	29.6 (26.8, 32.3)		1						
Yes (*n* = 127)	67	52.8 (44.0, 61.6)		2.88 (1.97, 4.23)	< 0.001					

a 95%CI: 95% confidence interval.

b χ2- Chi-square.

c Adjusted OR: adjusted odds ratio based on Binary Logistic Regression (Enter) using smartphone addiction in the baseline survey as a dependent variable and gender, parental education level, maternal education level, Body-Mass Index, and depression in the baseline survey as independent variables.

d Adjusted OR: adjusted odds ratio based on Binary Logistic Regression (Enter) using depression in the baseline survey as the dependent variable and gender, parental education level, maternal education level, Body-Mass Index, and smartphone addiction in the baseline survey as independent variables.

**Table 3:** The detection rates and predictors for smartphone addiction and depression in the following among respondents who responded to both surveys.

	**Smartphone addiction**					**Depression**				
	***N***	**Prevalence rates** ** (95%CI^**a**^)**	**χ2 ^**b**^ (*P* value)**	**Adjusted OR^**c**^** ** (95%CI)**	***P* value**	**N**	**Prevalence rates (95%CI^**a**^)**	**χ2 ^**b**^** ** (*P* value)**	**Adjusted OR^**d**^ (95%CI)**	***P* value**
Total (*n* = 1,186)	623	52.5 (49.7, 55.4)				335	28.2 (25.7, 30.8)			
**Gender**			5.25 (0.02)					12.27 (< 0.001)		
Male (*n* = 566)	317	56.0 (51.9, 60.1)		1.30 (1.03, 1.66)		187	33.0 (29.2, 36.9)		1.56 (1.20, 2.03)	
Female (*n* = 620)	306	49.4 (45.4, 53.3)		1	0.03	148	23.9 (20.5, 27.2)		1	< 0.001
**Parental education level**			0.86 (0.35)					0.87 (0.35)		
Below bachelor's degree (*n* = 924)	492	53.2 (50.0, 56.5)		1		267	28.9 (26.0, 31.8)		1	
Bachelor's degree or above (*n* = 262)	131	50.0 (43.9, 56.1)		1.01 (0.69, 1.46)	0.97	68	26.0 (20.6, 31.3)		1.14 (0.74, 1.74)	0.56
**Maternal education level**			1.09 (0.30)					1.23 (0.27)		
Below bachelor's degree (*n* = 1,016)	540	53.1 (50.1, 56.2)		1		293	28.8 (26.0, 31.6)		1	
Bachelor's degree or above (*n* = 170)	83	48.8 (41.2, 56.4)		0.94 (0.62, 1.43)	0.77	42	24.7 (18.2, 31.3)		0.97 (0.59, 1.58)	0.89
**Body-Mass Index**			4.04 (0.26)					2.55 (0.47)		
Wasting (*n* = 207)	101	48.8 (41.9, 55.7)		1		61	29.5 (23.2, 35.7)		1	
Normal (*n* = 720)	395	54.9 (51.2, 58.5)		1.28 (0.94, 1.76)	0.12	197	27.4 (24.1, 30.6)		0.86 (0.61, 1.22)	0.41
Overweight (*n* = 187)	91	48.7 (41.4, 55.9)		0.93 (0.62, 1.40)	0.72	60	32.1 (25.3, 38.8)		1.07 (0.69, 1.66)	0.78
Obese (*n* = 72)	36	50.0 (38.2, 61.8)		1.04 (0.60, 1.81)	0.88	17	23.6 (13.6, 33.7)		0.66 (0.35, 1.24)	0.20
**Smartphone addiction at follow-up**								16.06 (< 0.001)		
No (*n* = 563)						128	22.7 (19.3, 26.2)		1	
Yes (*n* = 623)						207	33.2 (29.5, 36.9)		1.65 (1.27, 2.14)	< 0.001
**Depression at follow-up**			16.06 (< 0.001)							
No (*n* = 851)	416	48.9 (45.5, 52.2)		1						
Yes (*n* = 335)	207	61.8 (56.6, 67.0)		1.64 (1.26, 2.13)	< 0.001					
**Smartphone addiction at baseline**										
No (*n* = 806)	368	45.7 (42.2, 49.1)								
Yes (*n* = 380)	255	67.1 (62.4, 71.9)								
**Depression at baseline**										
No (*n* = 1059)						258	24.4 (21.8, 27.0)			
Yes (*n* = 127)						77	60.6 (52.0, 69.2)			

a 95%CI: 95% confidence interval.

b χ2- Chi-square.

c Adjusted OR: adjusted odds ratio based on Binary Logistic Regression (Enter) using smartphone addiction in the follow-up survey as a dependent variable and gender, parental education level, maternal education level, Body-Mass Index, and depression in the follow-up survey as independent variables.

d Adjusted OR: adjusted odds ratio based on Binary Logistic Regression (Enter) using depression in the follow-up survey as the dependent variable and gender, parental education level, maternal education level, Body-Mass Index, and smartphone addiction in the follow-up survey as independent variables.

SA was more prevalent among males than in females, both at baseline and follow-up survey [aOR at baseline: 1.93 (95% CI: 1.49, 2.50); aOR at follow-up: 1.30 (95% CI: 1.03, 1.66)]. Depression was more prevalent among females at baseline [aOR: 1.73 (95% CI: 1.16, 2.56)]; but more prevalent among males at follow-up [aOR: 1.56 (95% CI: 1.20, 2.03)] (Tables 2, 3).

A strong cross-sectional association was observed between SA and depression for both the baseline and follow-up surveys. The adjusted OR (aOR) values after controlling for demographics were 2.88 (95% CI: 1.97, 4.23) and 1.65 (95% CI: 1.27, 2.14) for the baseline and follow-up surveys, respectively (Tables 2, 3).

### 3.3. The stability and new incidence of SA and depression over 12 months

Regarding the stability of SA and depression over 12 months, we observed that 67.1% (95% CI: 62.4%, 71.9%) of the participants in the baseline still scored over the SAS-SV threshold after 12 months; in addition, 60.6% (95% CI: 52.0%, 69.2%) of the participants in the cohort with baseline depressive symptoms still scored over the SDS threshold in the follow-up survey (Table 3).

Regarding the new incidence of SA and depression over 12 months, 45.7% (95% CI: 42.2%, 49.1%) of participants without baseline SA scored over the SAS-SV threshold after 12 months; in the follow-up survey, 24.4% (95% CI: 21.8%, 27.0%) of the participants without baseline depressive symptoms scored over the SDS threshold (Table 3).

### 3.4. The CLPM analysis between severities of SA and depression

**Figure 1:**
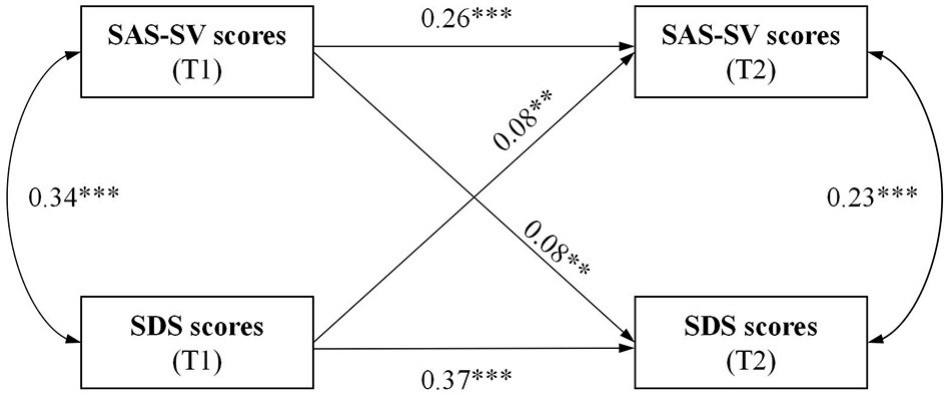
CLPM of SAS-SV and SDS scores at baseline (T1) and follow-up (T2, over 12 months) with standardized path coefficients. **P < 0.01; ***P < 0.001; T1 = assessed at baseline, T2 = assessed at follow-up. SAS-SV, Smartphone Addiction Scale-Short Version; SDS, Zung's Self-Rating Depression Scale; Single-headed arrows denote regressions, double-headed arrows denote correlations.

A well-fitted CLPM between severities of SA and depression was revealed by SEM analysis using continuous data, based on SAS-SV and SDS scores. The χ^2^ index (χ^2^*/df* ) was 32.16 (χ^2^ = 32.16, *df* =1, *P* < 0.001), CFI =0.94, GFI =0.99, and RMSEA = 0.16 (95% CI: 0.12, 0.21). All of these observed fit-indices reached the recommended cut-off values based on previous studies, except RMSEA. After adjusting for the effect of the covariates, although the severity of SA in the follow-up survey was predicted by baseline depression severity (path coefficient: 0.08; *P* = 0.008), the path coefficient was smaller than the predictive effect of the baseline SA severity relative to follow-up SA severity (0.26, *P* < 0.001) and the cross-sectional association between baseline SA severity and depression severity at baseline (0.34, *P* < 0.001) and follow-up (0.23, *P* < 0.001). Again, although depression severity in the follow-up survey was predicted by baseline SA severity (path coefficient: 0.08; *P* = 0.008), the path coefficient was smaller than that of the predictive effect of the severity of baseline depression to the severity of follow-up depression (0.37; *P* < 0.001) and cross-sectional associations between SA and depression in the baseline and follow-up surveys, respectively (Figure 1 and Table 4).

**Table 4:** Summary of the parameters for the whole sample, male and female of the CLPM.

	**All (*****n*** = **1,186)**				**Male (*****n*** = **566)**				**Female (*****n*** = **620)**			
	**B**	***SE***	***β***	***P***	**B**	***SE***	***β***	***P***	**B**	***SE***	***β***	***P***
SAS-SV-T1 to SAS-SV-T2	0.32	0.036	0.26	< 0.001	0.30	0.057	0.23	< 0.001	0.35	0.045	0.31	< 0.001
SAS-SV-T1 to SDS-T2	0.10	0.039	0.08	0.008	0.07	0.058	0.05	0.222	0.12	0.050	0.10	0.015
SDS-T1 to SAS-SV-T2	0.09	0.032	0.08	0.008	−0.01	0.053	−0.01	0.911	0.15	0.039	0.15	< 0.001
SDS-T1 to SDS-T2	0.45	0.035	0.37	< 0.001	0.50	0.054	0.37	< 0.001	0.44	0.044	0.39	< 0.001

SE, Standard error; T1 = assessed at baseline, T2 = assessed at follow-up. SAS-SV, Smartphone Addiction Scale-Short Version; SDS, Zung's Self-Rating Depression Scale. Standardized coefficients and standardized confidence intervals are shown.

### 3.5. Multi-group analysis by gender

**Figure 2:**
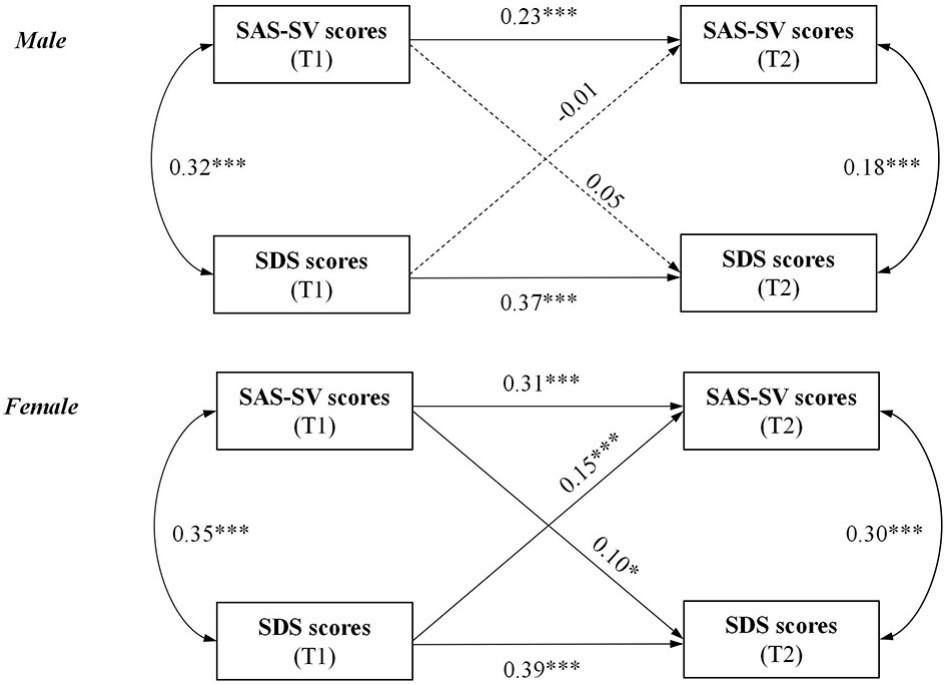
Path diagram of the CLPM by gender. *P < 0.05; **P < 0.01; ***P < 0.001; T1, assessed at baseline; T2, assessed at follow-up. SAS-SV, Smartphone Addiction Scale-Short Version; SDS, Zung's Self-Rating Depression Scale; Single-headed arrows denote regressions, double-headed arrows denote correlations; Solid lines present statistically significant associations and dashed lines present nonsignificant associations.

Multi-group analyses by gender of the CLPM was conducted, and the models revealed satisfactory model fit [male group: χ2/df = 15.08 (χ*2* = 15.08, *df* =1, *P* < 0.001), CFI = 0.93, GFI = 0.99, and RMSEA = 0.16 (95% CI: 0.09, 0.23); female group: χ*2/df* = 23.13 (χ*2* = 23.13, *df* =1, *P* < 0.001), CFI =0.93, GFI =0.98, and RMSEA = 0.19 (95% CI: 0.13, 0.26)]. Among the male group, the relationship between SAS-SV and SDS scores was not significant in either direction. In contrast, among the female group, baseline depression severity predicted the SA severity in the follow-up survey (path coefficient: 0.15; *P* < 0.001), and also, baseline SA severity predicted depression severity in the follow-up survey (path coefficient: 0.10; *P* = 0.015) (Figure 2 and Table 4).

## 4. Discussion

In this study, we adopted a two-wave longitudinal design and constructed a cross-lagged panel model (CLPM) model to examine the bidirectional relationship between SA and depression among freshmen. This study showed that, (1) high rates of SA and depression exist in the freshman cohort; (2) both SA and depression rates among freshmen are highly stable during the first year of college, while this period is also a high prevalence of SA and depression among baseline normal freshmen; (3) SA was significantly associated with depression in cross-sectional analyses at both time points; (4) CLPM showed that SA and depression were bidirectionally associated and this relationship was significant in the female group but not in the male group.

The rate of SA among incoming freshmen was 32.0%; at the time of follow-up, at the beginning of the sophomore year, this rate increased to 52.5%. This indicates that college freshmen may use their smartphones more and have a higher risk of SA. Of these, a significant number (67.1%) of students with baseline SA were still addicted to their smartphones at 12 months. This finding is important because some researchers have expressed doubts about the stability of SA and similar conditions when debating the validity of SA as a disorder (34, 35). Also, close to half of the students (45.7%) with non-SA at baseline developed SA symptoms at follow-up. Actually, the rate of SA (36, 37) among teenagers has risen dramatically during the COVID-19 pandemic. The incidence of SA among Italian adolescents has also increased, from 26.1% before the pandemic to 46.7% during the pandemic (36). Fund et al. (37) investigated a population of elementary school students in China and found that SA was significantly higher during the outbreak. Olvera et al. (38) indicated that TikTok saw a phenomenal increase in popularity during the COVID-19 pandemic, while Marengo et al. (39) found that TikTok was the most addictive application during this period. This also proves that it is important to guide and correct smartphone use behavior during the pandemic for college students, especially freshmen.

SA was more prevalent among males than females at baseline and follow-up surveys. It is inconsistent with previous studies, which suggested that females were significantly associated with a propensity for SA (34, 40, 41). We argued that such inconsistent findings might partially be attributed to the fact that the study population in this study was freshmen, that male students may be more proficient in using technology (42) and that there are some differences in the content of smartphone use between males and females (40, 43). Consistent with our results, a study conducted at an Indian medical school found that the SA rate reached 52.0% and was more prevalent in males than females (44). In addition, several other studies have found no significant gender differences in the prevalence of SA (45, 46). Further studies still need to unravel the inconsistent prevalence of SA in males and females.

As for depression, in our study, the prevalence of depression among freshmen was 10.7%, while one year later, this rate rose to 28.8%, and the new incidence during this period was 24.4%. On the one hand, this indicates that the prevalence of depression is high among Chinese adolescents. Duan et al. (13) indicated that the COVID-19 outbreak has had a significant psychosocial impact on Chinese adolescents and that high school graduates affected by the outbreak were significantly associated with depression. On the other hand, it points to a high prevalence of depression among freshmen. Ebert et al. (10) found that the incidence of major depressive disorder (MDD) within the first year of college was 6.9%, suggesting that the first year in college constitutes a risk period for the onset of MDD. Considering the context of life transition, freshmen are in an unfamiliar transition period and are vulnerable to depression and other mental health problems (47). During this period, students face a change in their living environment, a change in their learning methods, and a change from dependence to autonomy, and if they cannot adapt to this collective life in time, they can easily fall into emotional distress. As to gender, this is a controverted matter, with some studies suggesting that females are more likely to suffer from depression (48, 49), while in contrast, other researchers insist that males are more likely to experience depression than females (50). Our study found that the prevalence of depression was higher among freshmen females, and after 1 year, it was higher among males. A plausible explanation is that gender may have a significant effect only at the stage from moderate to severe depression (49), and the present study did not classify the severity level of depression. Therefore, the problem of depression among college students continues to require close attention and effort.

The CLPM results showed that the overall association between SA and depression was bidirectional; however, multi-group analysis by gender revealed that this bidirectional association persisted in the female group, whereas in the male group, the association was not significant in either direction. Among the female group, the cross-lagged effect coefficient from depression to SA was relatively larger than the reverse effect, suggesting that depressive symptoms may worsen SA to a greater extent. This echoes the previous findings, which suggested that SA or similar conditions can be caused by depressive symptoms (14, 51). Studies have found that depressed individuals derive less pleasure from social interactions and have increased sensitivity to social rejection (52, 53), but have a tendency to use social media more frequently (54). Frequent smartphone use as an avoidance-coping strategy appears to present them with a viable alternative to uncomfortable face-to-face contact in social situations. Furthermore, Elhai et al. (55) argued that smartphone use has also been proposed as a coping process for depressed mood (boredom tendencies, mood dysregulation, and pain intolerance). This avoidance-coping tends to foster reliance on online activities (52, 56). In the context of excessive smartphone use, not only may irrational beliefs or perceptions arise (feelings of inferiority, insecurity and self-esteem fluctuations) (57), but also social comparisons, a phenomenon of “emotional contagion” (58), leading to negative emotions such as depression and anxiety (59). Studies have also found that SA can lead to sleep disorders and delayed sleep, which are predictors of depression (60). Thus, SA and depression may form a vicious circle. Therefore, preventive control measures targeting depression should focus on smartphone use, while interventions targeting SA should incorporate a psychological component and appropriately increase attention to the female group.

This finding of gender differences is inconsistent with the studies of Park et al. (61), who observed significant changes in the longitudinal relationship between SA and depression among Korean adolescents across time, but no gender differences were found in the strength of these relationships. Possible mechanisms lie in physiological differences between the sexes (e.g., genetic vulnerability) (62), differences in self-concept (63), and differences in stress perception (64) that result in different emotional responses and behavioral patterns. Compared to males, females are more likely to perceive stress, and self-distraction may be considered one of the effective coping mechanisms (64). Females are more likely to internalize their negative emotions, whereas males' resort to externalizing behaviors, such as aggression and substance use (65, 66). Tang et al. (67) found that although female freshmen received more social support and had better help-seeking skills than males, females exhibited higher rates of depression. One possible explanation may be that females are perceived as more frequently, emotionally, and relatively exposed to stressful situations compared to males (68). Another reason could be that females are more likely to seek support through mobile social software, and their dependence on smartphones leads to a great sense of loss when they face real life (69). The findings suggest that researchers and practitioners have to take gender differences in understanding the bidirectional influences between SA and depression.

The current study has several limitations. Firstly, we recruited only freshmen from the same university to ensure consistent learning patterns. It may be possible to obtain richer results if college students in different years could be surveyed simultaneously at the same time. Therefore, further studies with a larger sample of representative Chinese college students are necessary to confirm our present findings. Secondly, only two-time points were assessed in this study. More waves and longer years of follow-up are warranted to understand better the stability of and changes in SA and depression. Third, the results of the validated factor analysis of SDS in this study were unsatisfactory, which we thought might be related to the fact that the study subjects were medical students with some medical background and were relatively familiar with the SDS scale. Attention should be paid to the selection of scales in future studies of depressive tendencies in college students, especially medical students. Fourth, other relevant questions, such as type of smartphone use, objective smartphone use time, and frequency of use, were not investigated, so we could not provide explicit confirmation of addicts' use patterns to support the discussion around this topic.

## 5. Conclusions

In conclusion, the study found that both smartphone addiction (SA) and depression were prevalent among freshmen and that there was a bidirectional predictive association between SA and depression, especially and only in the female group. Therefore, we should strengthen early intervention for behaviors related to SA among freshmen while focusing on related mental health issues.

## Data availability statement

The raw data supporting the conclusions of this article will be made available by the authors, without undue reservation.

## Ethics statement

The studies involving human participants were reviewed and approved by Anhui Medical University (No. 20190495). Written informed consent from the participants' legal guardian/next of kin was not required to participate in this study in accordance with the national legislation and the institutional requirements.

## Author contributions

KZ: conceptualization, formal analysis, writing–original draft, writing–review, and editing. HG: methodology, investigation, and data curation. TW: investigation, data curation, and formal analysis. JZ, GY, JR, XZ, and HY: investigation and data curation. XL, ZZhu, JD, HS, and GJ: investigation. JH, YS, and PS: validation and project administration. ZZha: validation and funding acquisition. All authors contributed to the article and approved the submitted version.
