# Supplementary material for: A bidirectional association between smartphone addiction and depression among college students: A cross-lagged panel model
Source: Front Public Health. 2023 Jan 24;11:1083856. doi: 10.3389/fpubh.2023.1083856 (PMC9902510; doi:10.3389/fpubh.2023.1083856)
Supplement: Supplementary file 1 [file Data_Sheet_1.PDF]

Supplementary materials

Supplemental Table 1. The original scale of the Smartphone Addition Scale Short Version (SAS-SV)

Items	Strongly disagree	Disagree	Weakly disagree	Weakly agree	Agree	Strongly agree
1. Missing planned work due to smartphone use.	1	2	3	4	5	6
2. Having a hard time concentrating in class, while doing assignments, or while working due to smartphone use.	1	2	3	4	5	6
3. Feeling pain in the wrists or at the back of the neck while using a smartphone.	1	2	3	4	5	6
4. Won't be able to stand not having a smartphone.	1	2	3	4	5	6
5. Feeling impatient and fretful when I am not holding my smartphone.	1	2	3	4	5	6
6. Having my smartphone in my mind even when I am not using it.	1	2	3	4	5	6
7. I will never give up using my smartphone even when my daily life is already greatly affected by it.	1	2	3	4	5	6
8. Constantly checking my smartphone so as not to miss conversations between other people on Twitter or Facebook.	1	2	3	4	5	6
9. Using my smartphone longer than I had intended.	1	2	3	4	5	6
10. The people around me tell me that I use my smartphone too much.	1	2	3	4	5	6

Supplemental Table 2. The Chinese version of the Smartphone Addition Scale Short Version (SAS-SV)

最近一年的实际情况	非常不符合	比较不符合	不符合	符合	比较符合	非常符合
1. 因使用智能手机而无法完成计划的工作。	1	2	3	4	5	6
2. 因使用智能手机，在上课、做作业或工作时很难集中精力。	1	2	3	4	5	6
3. 使用智能手机时，手腕或脖子后部感到疼。	1	2	3	4	5	6
4. 不能忍受没有智能手机。	1	2	3	4	5	6
5. 当智能手机不在手边时，会感到不耐烦和烦躁不安。	1	2	3	4	5	6
6. 即使不使用智能手机，我也时刻惦记它。	1	2	3	4	5	6
7. 即使智能手机已对我的日常生活造成巨大的影响，我也绝不放弃它。	1	2	3	4	5	6
8. 为了不错过社交软件（如微信、微博、QQ 等）的新信息，不断翻看智能手机。	1	2	3	4	5	6
9. 智能手机使用的时间超出了预期。	1	2	3	4	5	6
10. 身边的人都说我使用智能手机的时间太长了。	1	2	3	4	5	6

Supplemental Table 3. The original scale of the Zung's Self Rating Compression Scale (SDS)

	A Little of the Time	Some of the Time	Good Part of the Time	Most of the Time
1. I feel down-hearted and blue.	1	2	3	4
2. Morning is when I feel the best.	4	3	2	1
3. I have crying spells or feel like it.	1	2	3	4
4. I have trouble sleeping at night.	1	2	3	4
5. I eat as much as I used to.	4	3	2	1
6. I still enjoy sex.	4	3	2	1
7. I notice that I am losing weight.	1	2	3	4
8. I have trouble with constipation.	1	2	3	4
9. My heart beats faster than usual.	1	2	3	4
10. I get tired for no reason.	1	2	3	4
11. My mind is as clear as it used to be.	4	3	2	1
12. I find it easy to do the things I used to.	4	3	2	1
13. I am restless and can't keep still.	1	2	3	4
14. I feel hopeful about the future.	4	3	2	1
15. I am more irritable than usual.	1	2	3	4
16. I find it easy to make decisions.	4	3	2	1
17. I feel that I am useful and needed.	4	3	2	1
18. My life is pretty full.	4	3	2	1
19. I feel that others would be better off if I were dead.	1	2	3	4
20. I still enjoy the things I used to do.	4	3	2	1

Supplemental Table 4. The Chinese version of the Zung's Self Rating Compression Scale (SDS)

最近一个星期的感受	没有或很少时间	小部分时间	大部分时间	绝大部分或全部时间
1. 我觉得闷闷不乐，情绪低沉。	1	2	3	4
2. 我觉得一天中早晨最好。	4	3	2	1
3. 我一阵阵哭出来或觉得想哭。	1	2	3	4
4. 我晚上睡眠不好。	1	2	3	4
5. 我吃得跟平常一样多。	4	3	2	1
6. 我与异性密切接触时和以往一样感到愉快。	4	3	2	1
7. 我发现我的体重在下降。	1	2	3	4
8. 我有便秘的苦恼。	1	2	3	4
9. 我心跳比平常快。	1	2	3	4
10. 我无缘无故地感到疲乏。	1	2	3	4
11. 我的头脑跟平常一样清楚。	4	3	2	1
12. 我觉得经常做的事情并没有困难。	4	3	2	1
13. 我觉得不安而平静不下来。	1	2	3	4
14. 我对将来抱有希望。	4	3	2	1
15. 我比平常容易生气激动。	1	2	3	4
16. 我觉得作出决定是容易的。	4	3	2	1
17. 我觉得自已是个有用的人，有人需要我。	4	3	2	1
18. 我的生活过得很有意思。	4	3	2	1
19. 我认为如果我死了，别人会生活得好些。	1	2	3	4
20. 平常感兴趣的事我仍然照样感兴趣。	4	3	2	1
